# Differences in Cortical Structure and Functional MRI Connectivity in High Functioning Autism

**DOI:** 10.3389/fneur.2018.00539

**Published:** 2018-07-10

**Authors:** Alessandra M. Pereira, Brunno M. Campos, Ana C. Coan, Luiz F. Pegoraro, Thiago J. R. de Rezende, Ignacio Obeso, Paulo Dalgalarrondo, Jaderson C. da Costa, Jean-Claude Dreher, Fernando Cendes

**Affiliations:** ^1^Neuroimaging Laboratory, School of Medical Sciences, The Brazilian Institute of Neuroscience and Neurotechnology, University of Campinas, Campinas, Brazil; ^2^Department of Pediatrics, Pontifícia Universidade Católica do Rio Grande do Sul, Porto Alegre, Brazil; ^3^Department of Psychiatry, State University of Campinas, Campinas, Brazil; ^4^Center for Cognitive Neuroscience, Reward and Decision Making Group, Centre National de la Recherche Scientifique, UMR 5229, Lyon, France; ^5^Centro Integral en Neurociencias A.C., Hospital HM Puerta del Sur en Madrid, Madrid, Spain; ^6^Brain Institute (InsCer), Pontifícia Universidade Católica do Rio Grande do Sul, Porto Alegre, Brazil

**Keywords:** autism spectrum disorders, functional connectivity, MRI, cortical thickness, default mode network (DMN), social communication, stereotyped behavior

## Abstract

Autism spectrum disorders (ASD) represent a complex group of neurodevelopmental conditions characterized by deficits in communication and social behaviors. We examined the functional connectivity (FC) of the default mode network (DMN) and its relation to multimodal morphometry to investigate superregional, system-level alterations in a group of 22 adolescents and young adults with high-functioning autism compared to age-, and intelligence quotient-matched 29 healthy controls. The main findings were that ASD patients had gray matter (GM) reduction, decreased cortical thickness and larger cortical surface areas in several brain regions, including the cingulate, temporal lobes, and amygdala, as well as increased gyrification in regions associated with encoding visual memories and areas of the sensorimotor component of the DMN, more pronounced in the left hemisphere. Moreover, patients with ASD had decreased connectivity between the posterior cingulate cortex, and areas of the executive control component of the DMN and increased FC between the anteromedial prefrontal cortex and areas of the sensorimotor component of the DMN. Reduced cortical thickness in the right inferior frontal lobe correlated with higher social impairment according to the scores of the Autism Diagnostic Interview-Revised (ADI-R). Reduced cortical thickness in left frontal regions, as well as an increased cortical thickness in the right temporal pole and posterior cingulate, were associated with worse scores on the communication domain of the ADI-R. We found no association between scores on the restrictive and repetitive behaviors domain of ADI-R with structural measures or FC. The combination of these structural and connectivity abnormalities may help to explain some of the core behaviors in high-functioning ASD and need to be investigated further.

## Introduction

Autism spectrum disorders (ASD) represent a complex group of neurodevelopmental conditions characterized by deficits in social behaviors, including both interpersonal social processes and self-referential thought (1). This condition is reported to affect 1 in 59 individuals according to the last CDC update of autism's estimated prevalence (2). The pathology of ASD is currently considered a disruption of brain development time-course with a wide range of heterogeneity among patients (3). The specific neurobiological substrates of this lifelong developmental disability remain unclear. Several studies reported a combination of structural abnormalities along with atypical brain connectivity in ASD (4–15). These abnormalities could help explain some of the symptoms of ASD and their severity.

Early investigations in ASD showed an increase in total brain volume at 2–4 years of age persisting into childhood but not adolescence (16). Some areas increase more than others, including frontal and temporal regions and the amygdala, while other structures present reduction in volume, such as the corpus callosum (17–26), probably indicating dysfunction of intra- and inter-hemispheric connectivity (15, 27–36). The first generation of studies using brain imaging failed to report consistent localized neocortical brain dysfunction (37, 38). However, structural neuroimaging has indicated various sites of anatomical abnormalities, providing some clues for a better understanding of this condition (17, 39–44).

Despite some inconsistencies, there is a trend from more recent studies which have observed regional increases of gray matter (GM) accompanied by local reductions of white matter (WM) (6, 38, 45). These findings support an increased local but reduced long-distance cortico-cortical reciprocal activity and functional coupling (46–48). Converging lines of evidence suggest that ASD is a complex disorder of brain connectivity (49, 50), involving aberrant functional connectivity (FC) within the default mode network (DMN), as well as between the DMN and several cortical and subcortical areas (13, 15, 27, 30–36, 44, 51–70, 107, 135).

**Figure 1:**
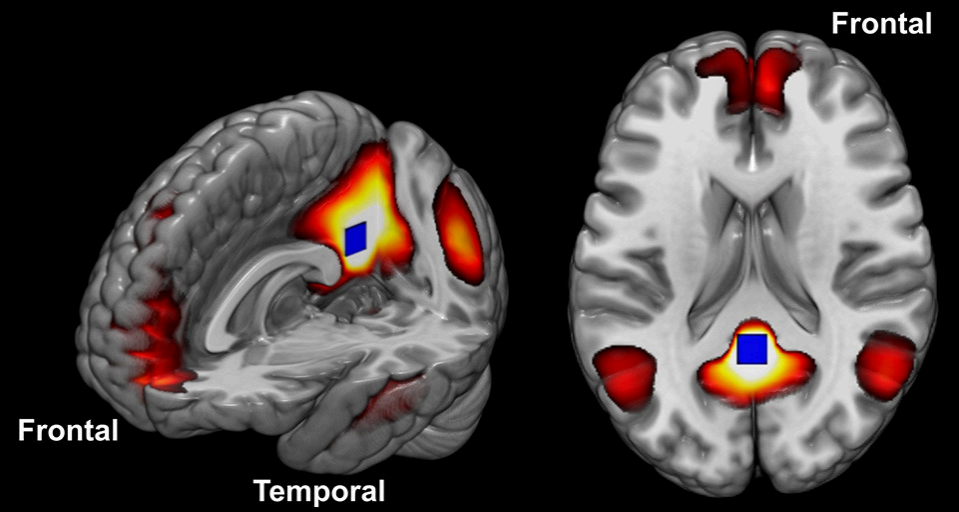
The DMN constituent components. The blue square placed in the posterior cingulate cortex illustrates the seed position described in the methods.

The DMN is a set of structures known to be particularly engaged when participants are at rest (Figure 1). Anatomically, this network consists of the posterior cingulate cortex (PCC), retrosplenial cortex, lateral parietal/angular gyrus, medial prefrontal cortex, superior frontal gyrus, regions of the temporal lobe, and the parahippocampal gyrus (54, 71–73, 79). Many have speculated that the DMN function may extend beyond cognitive processes and encompass the role of maintaining homeostasis between excitatory and inhibitory neuronal responses (74, 75). Others have argued that it is active when contemplating scenarios and events, when the mind is wandering, or when conducting lower-level observations of the individual's external surroundings (76–79). More recently, the “developmental disconnection model,” proposed by many authors, links the core symptoms of ASD to weak FC between remote cortical regions and an excess of FC within local regions (80–82). For recent reviews in the topic see references (6, 15, 37, 43, 44, 50, 57, 67, 83–86).

It is currently unclear the extent of regions overlap between abnormal structural and functional connectivity in ASD patients and its relationship with different clinical presentations in the spectrum of this condition (26, 87, 88). The understanding of the relationship between structural and functional alterations is also compromised by the high heterogeneity of individuals and the age-related differences reported among different ASD groups (26). The comparison between brain structure and function in a single group of ASD individuals with similar phenotypic pattern can shed light on these complex interactions and establish a link with clinical symptomatology in these patients.

We aimed to characterize the relationships between structural and functional abnormalities in a cohort of patients with high-functioning autism. We performed a high resolution multimodal structural (cortical thickness, gyrification index, surface area and GM volume) and functional (resting-state FC) analysis to detect superregional, system-level alterations attempting to establish a neurobiological foundation to pathology and clinical symptoms in this part of the spectrum of autism—adolescents and young adults with high-functioning autism without associated depression, psychosis, seizures, or other major psychiatric disorders.

## Methods

### Participants

We recruited 22 adolescents and young adults with ASD and 29 normal controls from the local community and the University of Campinas. This study was approved by the Ethics Committee of the University of Campinas (plataformabrasil.saude.gov.br; reference number: CAAE 02388012.5.0000.5404; number of the approved ethical statement: 190409). All participants provided written informed consent approved by the Ethics Committee. For the participants younger than 18 years of age, we obtained informed consent from parents or guardians, as well as from the participants themselves.

A trained and qualified clinician made the diagnosis of ASD using the DSM-5 criteria after interviewing the family and examining each patient. A second investigator confirmed the diagnosis using the “Current” Scores of the Autism Diagnostic Interview-Revised (ADI-R) (89). The ADI-R is a clinical diagnostic instrument for assessing autism in children and adults (89). The ADI-R provides a diagnostic algorithm for autism as described in both the ICD-10 and DSM-IV and is one of the most important validated ASD measures available in Brazil. The clinician's observation provides the opportunity to put the patient's behavior into the context of knowledge about other patients, but information from caregivers provides a broader context needed in understanding the patient's day to day behavior in a wide range of situations, his or her history, as well as family expectations, resources, and experiences and other important contextual factors. Thus, patient's testing and parent interviews should be viewed as complementary and necessary components of the diagnostic evaluation after the clinical evaluation and DSM-5 criteria. All patients were required to have a full-scale IQ greater than 85, as measured by the Wechsler Abbreviated Scale of Intelligence.

Exclusion criteria comprised a history of major psychiatric disorders (e.g., depression, psychosis), seizure, head injury, toxic exposure, facial dysmorphic features, and the evidence of genetic, metabolic, or infectious disorders. We also excluded individuals with secondary autism related to a specific etiology such as tuberous sclerosis or Fragile X syndrome (all included patients had a negative investigation of tuberous sclerosis and Fragile X syndrome).

Thirteen individuals in the ASD group were using a variety of psychoactive medications. Nine subjects were not under psychoactive drug treatment. Five subjects were taking psychostimulants, seven were taking antipsychotics, and six were taking selective serotonin reuptake inhibitors (SSRIs) for anxiety and compulsive behaviors. Six of these subjects were using more than one of the medications listed above. Participants were instructed not take any medication 1 day before their visit.

### Neuroimaging data acquisition

We acquired functional and structural MRIs on a 3T scanner (Phillips, Achieva; Best, The Netherlands) with the following protocol:

– Resting-state fMRI: 6 min echo-planar images (EPIs), 180 dynamics, voxel size = 3 × 3 × 3 mm^3^, 40 slices, no gap, FOV = 240 × 240 × 120 mm^3^, TE = 30 ms, TR = 2,000 ms, flip angle = 90°. For this specific acquisition, we instructed all individuals to keep their eyes closed, not to fall asleep and try not to move for the duration of the scan. We used memory foam pillows placed around the participant's head to minimize head movement.
– Structural MRI: Volumetric T1-weighted images acquired on the sagittal plane, voxel size = 1 × 1 × 1 mm^3^, no gap, TR = 7 ms, TE = 3.2 ms, flip angle = 8°, FOV = 240 × 240 × 180 mm^3^. The number of slices varies with the size of the head, with an average of 160 sagittal slices.

MRI sequences were corrected for gradient non-linearity during the reconstruction step in the Phillips scanner. We performed a visual inspection of all structural and functional images to assess image quality, movement artifacts, and the existence of clinically relevant abnormalities.

### Image processing and analysis

Our MRI phenotyping combined group- and individual-level analysis of GM volume, cortical thickness and folding complexity, which are three established *in vivo* markers of brain morphology and development. There was no difference between the groups on movement in the scanner for the structural imaging.

#### Voxel-based morphometry analysis

We performed VBM with the VBM8/SPM8 toolbox (Wellcome Department of Cognitive Neurology, http://www.fil.ion.ucl.ac.uk) for detection of GM volume abnormalities. VBM allows the automated identification of the whole brain GM differences between groups (90). Post-processing of the T1-weighted images included normalization to the same stereotaxic space (MNI-152 template), modulation and segmentation of the images into GM, WM and cerebrospinal fluid (CSF). The DARTEL algorithm was included to increase the accuracy of the alignment between subjects (91). The resultant GM images were smoothed with a 10 mm FWHM isotropic Gaussian kernel. We excluded eight outliers (four ASD patients and four controls) detected in a quality test for image homogeneity and co-registration. Therefore, the final VBM analysis included 19 ASD patients and 25 controls (all other analyses from here on included the 22 patients and 29 controls).

We used two-sample *t*-tests (to adjust for multiple comparisons we considered a *p* < 0.001, minimum of 30 contiguous voxels) to search for areas of volume reduction or increase in ASD patients. First, we looked for areas of GM volume reduction or increase in the ASD group with age as covariable. As a second approach, we looked at the differences between groups in the correlation between age and GM volumes with total IQ as a covariate.

### Cortical thickness, surface area, and gyrification analysis with FreeSurfer

We performed cortical reconstruction and volumetric segmentation with the FreeSurfer image analysis suite (http://surfer.nmr.mgh.harvard.edu/), which is a well-validated method already described in previous publications (92–94). A single filled WM volume was generated for each hemisphere after intensity normalization, skull stripping, and image segmentation using a connected components algorithm. A surface tessellation was generated for each WM volume by fitting a deformable template. This resulted in a triangular cortical mesh for GM and WM surfaces in each hemisphere. Cortical thickness, then, was calculated as the shortest distance between GM and WM surfaces. Vertex-wise measurements of surface area were determined as the area of a vertex on the GM surface (5). We used the FreeSurfer default Gaussian filter of 10 mm FWHM to smooth the surfaces (92, 94).

Another volumetric measure obtained from FreeSurfer is the local gyrification index (LGI) which was developed by Schaer et al. (95). The LGI was defined as the ratio between the GM surface border and an outer border in successive coronal sections (96). To calculate this LGI, FreeSurfer uses both tessellated outer and inner contours of the pial surface, which were covered by a triangle mesh. For each vertex on the outer surface, a spherical region of interest is created with a standard size of 25 mm radius. Therefore, the LGI is given as the ratio between the outer area on the surface and the area comprehended in the real pial surface (95). Thus, the LGI for each vertex on the pial surface reflects the amount of cortex buried in its locality. The LGI values obtained were mapped onto a normalized cortical surface.

We then compared regional cortical thickness, surface area and gyrification index between autism and control groups using a general linear model (GLM) with age and total IQ as covariates. To correct for multiple comparisons, we performed a cluster-based correction (level of significance at α = 0.01) (97).

### ROI analysis with data extracted from FreeSurfer

ROI measures of cortical thickness, cortical area and LGIs for 33 gyral regions generated by FreeSurfer (98, 99) (https://surfer.nmr.mgh.harvard.edu/fswiki/FsTutorial/AnatomicalROI#Groupstatsfiles) were corrected for total intracranial volume generated by FreeSurfer and exported to SPSS Statistics version 20 (IBM Corp. Released 2011. IBM SPSS Statistics for Windows, Version 20.0. Armonk, NY: IBM Corp.).

Group differences in gyral-level cortical thickness, cortical area, and LGIs were analyzed using mixed GLMs with diagnosis (autism vs. controls) as the between-subjects factor, the 33 gyral regions from both hemispheres (98, 99) as the within-subjects factors, also with age and total IQ as covariates. We also ran the same mixed GLM for subcortical volumes generated by FreeSurfer. All comparisons between controls and patients were Bonferroni corrected for multiple comparisons.

#### Resting-state functional MRI processing and analysis

To perform the resting-state processing and analysis, we used the UF^2^C (User-Friendly Functional Connectivity; https://www.lniunicamp.com/uf2c) toolbox (100) on a PC running MATLAB 2013a (The MathWorks, Inc., Natick, MA, USA) with SPM8 (Wellcome Trust Centre for Neuroimaging). The UF2C toolbox (100) pipeline started with a standard image preprocessing protocol which includes: (i) functional realignment to the mean image (movement parameters are saved); (ii) structural-functional co-registration; (iii) structural segmentation into GM, WM and CSF tissues; (iv) functional and structural normalization (MNI 152); (v) functional image smoothing (kernel with double voxel sizes = 6 × 6 × 6 mm^3^).

We used the functional and structural T1-weighted images of all subjects as data input. The GM, WM, and CSF maps were spatially adjusted (sinc interpolation [or Whittaker–Shannon interpolation] of third degree) to the functional image, aiming to obtain functional segmented maps (GM, WM, and CSF). A multilinear regression was performed including WM and CSF global signal fluctuations and six movement parameters (three translational and three rotational) to reduce their confounding influence on the GM signal (101). Subsequently, a band-pass filter (0.008–0.1 Hz) was applied to remove low-frequency drifts and artifacts arising from cardiac or respiratory rate (102).

To reduce the chance of false positives/negatives, we controlled the amount of motion during scanning sessions using a cumulative value of movement equal or higher than 3 mm (size of one voxel) using the first volume as a reference as the cut-off to exclude subjects from the analysis. One patient was excluded from the resting-state analyses due to excessive movements during the fMRI acquisition. There was no difference between groups in the amount of movement during the scans: multivariate general linear model, Tukey's corrected with maximum displacement on axes X (controls average 0.77 mm ±0.47; patients average 0.78 ± 0.49), Y (controls average 0.30 mm ± 0.1; patients average 0.41 ± 0.22), and Z (controls average 1.15 mm ± 0.47; patients average 1.35 ± 0.51), average framewise displacement (controls average 0.18 mm ± 0.04; patients average 0.24 ± 0.06), and derivative variance (DVAR) (control average 3.18% *SD* ± 0.40; patients average 3.22% *SD* ± 0.36) were added as variables.

We estimated the cross-correlations using a cubic seed (9 × 9 × 9 mm3) to extract the reference time-series (64). The reference time-series was correlated with each gray matter voxel creating the correlation maps. We varied the seed position according to the analysis described below.

#### DMN analysis

The motivation to investigate the connectivity of the whole brain to and from the DMN came from the fact that: (a) it is a very stable and reproducible network (103, 104), (b) several studies have shown alterations in the DMN in ASD, including high functioning autism (88), and (c) it connects to most regions of the brain, and in particular, to regions processing salience, attention, and negative affect (105). To study the DMN, we positioned the seed on the PCC (centered on the MNI coordinate −41 13 −29) because this is one of the most active areas within the DMN, and it is possible to place a seed region involving both hemispheres at once (the blue square in Figure 1 illustrates the position of this seed). We used the standard seed-based FC methodology, in which the whole averaged time series of the seed region is used as a reference to calculate the correlation with the GM voxels. We performed these steps individually generating a 3D r-score map for each volunteer. We converted all individual r-score maps resultant from the connectivity analysis to z-scores (Fisher's transformation) and performed a spatial smoothing (6 × 6 × 6 mm^3^ FWHM), aiming to reduce high discrepancies in neighbor voxels.

#### Other seed positions

Additional to the seed positioned in the PCC (from the DMN), we tested other four seeds that we judged relevant for ASD verbal communication and social skills, according to findings from previous publications (6, 37, 106): (i) bilateral medial frontal region (MNI 0 49 −3); (ii) left + right amygdala (MNI −23 −4 −20); (iii) left anterior hippocampus (MNI −24 −13 −20); (iv) left temporal pole (−41 13 −29). We used the same steps as described for the generation of the 3D r-score *DMN* maps to obtain individual 3D statistical maps for the functional connectivity maps derived from seeds in these four positions. We did not include seeds in other areas also considered important for ASD, such as the caudate, to avoid too many comparisons and to focus mainly on regions more directly related to emotional communication and interpersonal interactions.

The functional connectivity preprocessing was developed aiming to avoid possible confounding effects raised from structural variations. The functional images were segmented using the tissues probabilistic maps obtained from the T1WI, with consistent thresholds. This means that the resultant post-processed functional images included only voxels with the upper threshold probability to be GM or GM/WM. Additionally, the seeds time series extraction applied an algorithm that excludes by the average time series, voxels with a temporal behavior that is considered a minor outlier regarding the others. These last steps exclude from the seed, voxels which are functionally discrepant (see Supplementary Image 1).

As in the previous section, all individual r-score maps resultant from the connectivity analysis to z-scores (Fisher's transformation) and performed a spatial smoothing (6 × 6 × 6 mm^3^ FWHM), aiming to reduce high discrepancies in neighbor voxels. We applied a two-sample *t*-test (to adjust for multiple comparisons we considered a *p* < 0.001, with a minimum of 10 contiguous voxels) with age added as covariate to compare controls and patient's groups resulting in two t-maps: a map showing areas that were more functionally connected in controls than in patients and a map showing the opposite.

### Correlations with the clinical phenotype

We explored how the neuroanatomical and functional differences observed in the ASD group may be related to the clinical outcome. For that purpose, we conducted multiple correlation analyses between the ROI measures of cortical thickness, cortical area, and LGIs for the 33 gyral regions of each hemisphere generated by FreeSurfer (98, 99) and values from the PCC seed-based functional connectivity analysis (Resting-state analysis) vs. the “Current” Scores obtained at the ADI-R (scores in each of the three content areas: communication and language, social interaction, and restricted, repetitive behaviors), with age and total IQ as covariates and with Bonferroni correction for multiple comparisons using SPSS Statistics version 20 (IBM Corp. Released 2011. IBM SPSS Statistics for Windows, Version 20.0. Armonk, NY: IBM Corp.).

### Analyses of overlapping of abnormalities across modalities

We analyzed the number of voxels that coincided with the resting-state fMRI and structural analyses using co-registration of statistical maps. This procedure was automated and based on the maps matrix intersection, providing relative percentages of overlapping among maps. Maps with distinct resolution were interpolated using 4th degree B-Spline interpolation.

In addition, we also investigated if the areas of abnormalities were near or within the same anatomical sub-region by sub-region by atlas labeling coincidence.

## Results

### Subject demographics and global brain measures

There were no significant differences in age between ASD (*n* = 22; mean ± *SD*: 17.45 ± 3.29) and controls (*n* = 29; 18.48 ± 2.82, two-sample *t*-test, *p* = 0.24). There was no significant difference in sex ratios between groups (Fisher's exact test; *p* = 0.22). We found no significant differences in full scale and performance IQ (*p* = 0.1) but, as expected, the ASD group displayed significantly lower verbal scale IQ (*p* = 0.03; see Table 1). There were also no significant between-group differences in total brain volume or total surface area (*p* > 0.05).

**Table 1:** Summary of clinical data.

	**Controls (*n* = 29)**	**ASD (*n* = 22)**
Age (range)	18.48 ± 2.82 SD (14–25)	17.45 ± 3.29 SD (14–25)
Sex	19M:10F	18M:4F
Handedness Rt to Lt	28:1	19:3
Full scale IQ (range)	105.83 ± 9.64 (90–127)	99.77 ± 9.5 (87–121)
Performance IQ (range)	107.79 ± 11.91 (86–128)	101.77 ± 12.25 (84–129)
Verbal IQ^*^ (range)	103.86 ± 9.53 (87–123)	98.95 ± 9.67 (85–124)
ADI-R social (range)	–	20.50 ± 5.38 (10–29)
ADI-R communication (range)	–	13.82 ± 4.36 (6–21)
ADI-R repetitive behavior (range)	–	6.50 ± 1.78 (3–10)

*ADI-R, Autism Diagnostic Interview-Revised (“Current” Scores); ASD, autism spectrum disorder. There were no significant differences between the ASD and control groups in age, full IQ and performance IQ at p < 0.05 (two-tailed). There was no significant difference in sex ratios between groups (Fisher's exact test; p = 0.22)*.

* *There was a significant difference in verbal IQ (p = 0.03). The following cutoff scores were used: ADI-R social, greater than 10; communication, greater than 6; and repetitive behavior, greater than 3. Rt to Lt, right to left ratio*.

All imaging analyses were covaried for age and total IQ and corrected for multiple comparisons as described in the methods.

### Voxel-based morphometry (VBM) analysis

**Figure 2:**
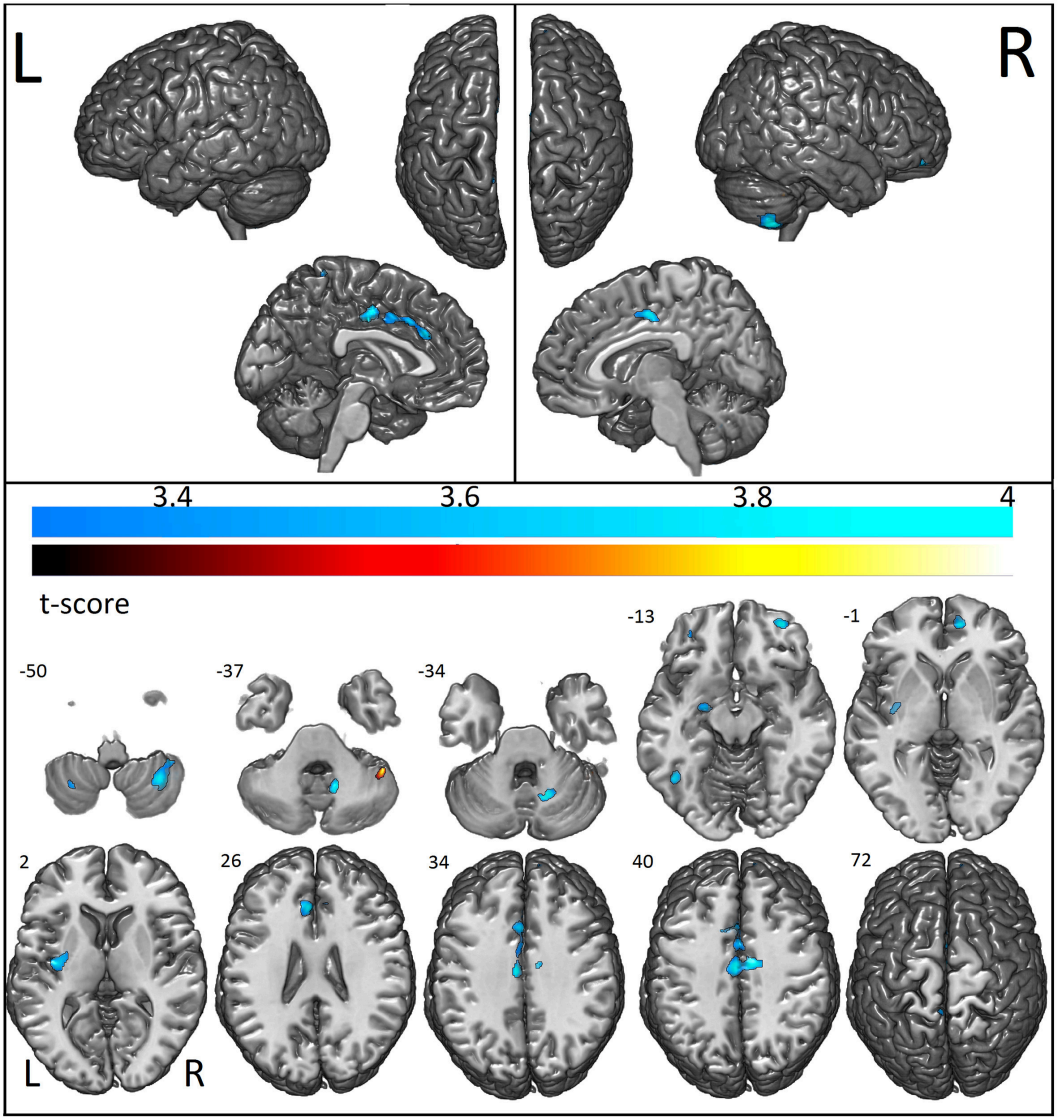
Areas with decreased (cool colormap) and increased (hot colormap) cortical voxel-based morphometry in patients when compared to controls. In shades of blue (cool colormap), the most significant regions with decreased gray matter (voxel-based morphometry, two sample *t*-test, *p* < 0.001, cluster with at least 30 voxels) in patients compared to controls. In the hot colormap (black to yellow), regions of increased gray matter (voxel-based morphometry, two sample *t*-test *p* < 0.001 clusters with at least 30 voxels).

VBM showed that individuals with ASD had reduced GM concentration in the cerebellum bilaterally (right anterior and posterior lobe and left posterior lobe), bilateral anterior cingulate, right middle, medial, and superior frontal gyrus, left fusiform gyrus, parahippocampus, amygdala, paracentral, and postcentral gyrus and claustrum. Increased GM concentration was detected in the right cerebellum and brainstem (Figure 2; Table 2).

**Table 2:** Areas of reduced gray matter concentration and increased gray matter concentration by VBM in patients with ASD in comparison with a group of healthy individuals.

**Voxels**	**Area**	**Side**	**T score**	**MNI Coordinates**
**AREAS OF REDUCED GRAY MATTER vbm CONCENTRATION IN PATIENTS WITH ASD**				
1804	Cerebellum, Posterior lobe	Right	4.24	33 −55 −53
259	Fusiform gyrus	Left	4.58	−44 −54 −8
347	Cerebellum, Anterior lobe	Right	4.45	14 −60 −30
1562	Cingulate gyrus	Left	4.43	−6 −13 37
	Cingulate gyrus	Right	4.25	8 −9 42
	Paracentral lobule	Left	4.25	−8 −9 45
263	Middle frontal gyrus	Right	4.42	32 53 −14
642	Claustrum	Left	4.12	−38 −10 3
170	Medial frontal gyrus	Right	4.06	12 51 1
66	Parahippocampal gyrus	Left	3.89	−15 −18 −26
121	Lentiform nucleus	Left	3.85	−18 −9 −9
	Amygdala	Left	3.74	−26 −7 −14
73	Postcentral gyrus	Left	3.72	−6 −42 70
77	Cerebellum, Posterior lobe	Left	3.69	−30 −58 −48
37	Superior frontal gyrus	Right	3.57	12 60 30
32	Cingulate gyrus	Right	3.49	18 33 22
**AREAS OF INCREASED GRAY MATTER vbm CONCENTRATION IN PATIENTS WITH ASD**				
96	Cerebellum, Posterior lobe	Right	3.93	45 −45 −38
42	Brainstem	Left/right	3.52	−2 −37 −27

In a correlation between age and GM volumes (i.e., areas with decreased GM volume in patients with increasing age as compared to controls), we observed that ASD participants had more age-related GM atrophy than controls exclusively in the left temporal lobe (temporal pole, middle temporal gyrus, parahippocampal gyrus, uncus) (*p* < 0.001, Supplementary Image 2; Table 3).

**Table 3:** Areas with significant gray matter VBM reduction influenced by the age in patients with ASD.

**Voxels**	**Area**	**Side**	**T–score**	**MNI Coordinates**		
378	Middle Temporal Gyrus	Left	3.98	−45	6	−36
112	Parahippocampal	Left	3.65	−21	−10.5	−34.5
78	Uncus/Amygdala	Left	3.88	−33	−10.5	−37.5
67	Superior Temporal Sulcus/Gyrus	Left	3.52	−63	−34.5	13.5

*p < 0.001; cluster with at least 30 voxels. All these four areas had significantly reduced functional connectivity on the seed analysis (see Table 6)*.

### Cortical thickness and gyrification index using FreeSurfer

#### Vertex-by-vertex analysis

**Figure 3:**
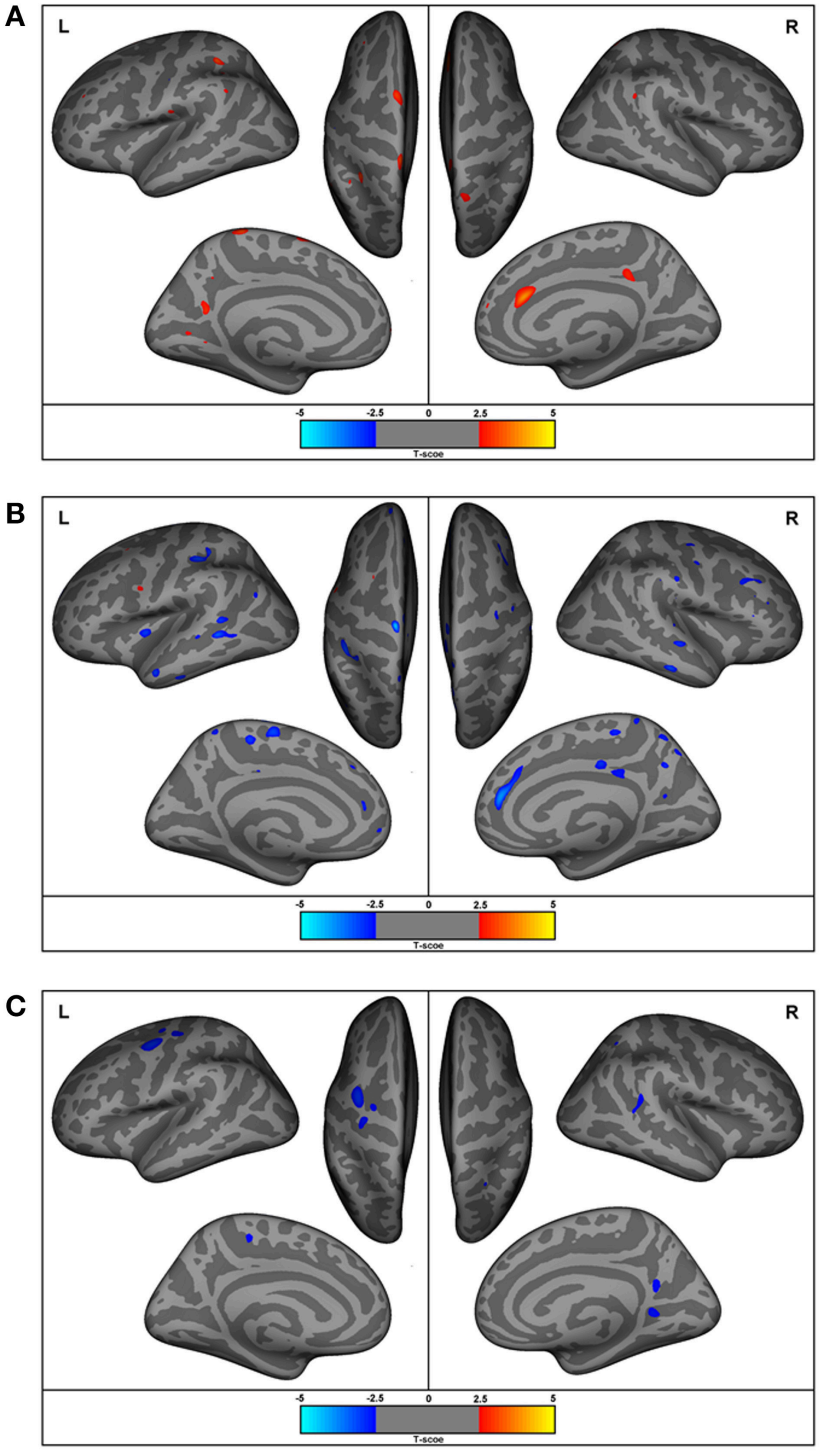
Regions with differences in cortical thickness, surface areas, and gyrification. The most significant clusters for group analysis using a GLM vertex-wise approach, between control and autism groups for left and right hemispheres. In red are areas of decreased and in blue are areas of increased values in patients with autism. All results were corrected for multiple comparisons (Cluster-based correction). **(A)** ASD presented decreased (in red) cortical thickness in the right cingulate, precentral, superior frontal, superior, and inferior parietal regions. In the left hemisphere, decreased cortical thickness was observed in the supramarginal, superior parietal, paracentral, precuneus, superior, and middle frontal and lingual gyrus, and increased thickness in the postcentral area. **(B)** Increased surface areas in the superior and middle frontal and precuneus (coinciding with the regions with reduced cortical thickness), as well as in the pre- and post-central, orbitofrontal, posterior cingulate, inferior parietal, temporal lobe (superior, middle, inferior temporal), and insular regions in ASD. **(C)** Increased gyrification in the lingual, precuneus, superior temporal sulcus, and superior parietal areas in the right hemisphere and the precentral and paracentral areas of the left hemisphere.

Individuals with ASD presented decreased cortical thickness in the right hemisphere over the cingulate, precentral, superior frontal, superior, and inferior parietal regions. In the left hemisphere, decreased cortical thickness was observed in the supramarginal, superior parietal, paracentral, precuneus, superior, and middle frontal and lingual gyrus (the areas of decreased cortical thickness are shown in red in Figure 3A), and increased thickness was observed in the postcentral area (Table 4).

**Table 4:** Areas of decreased cortical thickness by FreeSurfer vertex-wise analysis in patients with ASD.

**Cluster**	***p*-value**	**X**	**Y**	**Z**	**Vertex**	**Anatomical region**	**Macro anatomical region**
		**MNI Coordinates**					
**LEFT HEMISPHERE**							
1	0.001	−36.5	−43.67	9.21	58690	Inf. Supramarginal G	Supramarginal
2	<0.001	−21.47	−68.69	12.95	146808	Superior Temp S	Superior temporal sulcus
3	0.001	−13.68	−18.6	52.44	41967	Postcentral G	Postcentral
4	0.004	−0.05	−53.7	47.49	81380	Intraparietal S	Superior parietal
5	0.004	−13.64	−88.19	−5.07	101248	Middle occipital G	Occipital
6	0.001	8.59	11.93	64.19	44865	Sup. part of precentral S	Precentral
7	0.002	−23.88	−69.23	−38.08	69991	Inferior temporal S	Temporal
8	0.003	16.3	−65.67	53.17	53854	Superior parietal G	Superior parietal
9	0.001	28.43	−65.42	20.57	64736	Precuneus G	Precuneus
10	0.001	28.96	−12.24	53.54	26765	Sup. Frontal G	Paracentral
11	0.002	27.57	42.06	56.47	152760	Sup. Frontal G	Superior frontal
12	0.003	−6.05	96.89	−21.68	58366	Middle frontal G	Rostral middle frontal
**RIGHT HEMISPHERE**							
1	0.004	8.66	19.20	50.53	29786	Sup. part of precentral S	Precentral
2	0.003	20.9	72.25	−1.21	4081	Sup. frontal G	Superior frontal
3	0.002	−10.97	7.58	66.56	5767	Precentral G	Precentral
4	0.005	−9.49	89.20	−46.04	119767	Orbital G	Pars orbitalis
5	<0.001	−30.83	17.08	42.62	108535	Postcentral G	Postcentral
6	0.001	−26.24	−73.11	12.69	45913	Sup. temporal S	Superior Temporal sulcus
7	0.001	−26.08	28.49	63.48	144822	Central S	Precentral

*Level of significance equal to 0.01. All results were corrected for multiple comparisons (cluster-based correction). S, sulcus; G, gyrus*.

The ASD group had increased cortical surface in the following areas in the right hemisphere: cingulate, precentral, and superior frontal regions (which coincided with regions with decreased cortical thickness), as well as middle frontal, pars triangularis, supramarginal, precuneus, paracentral, superior, and middle temporal, and lateral occipital regions. In the left hemisphere, the ASD group had increased surface areas in the superior and middle frontal and precuneus (coinciding with the regions with reduced cortical thickness), as well as in the pre- and post-central, orbitofrontal, posterior cingulate, inferior parietal, temporal lobe (superior, middle, and inferior temporal regions) and insular regions (Figure 3B).

Gyrification was increased in the lingual, precuneus, superior temporal sulcus and superior parietal areas in the right hemisphere, and in the precentral and paracentral areas of the left hemisphere (Figure 3C).

#### Region of interest (ROI) analysis with FreeSurfer data

When examining gyral-based differences in cortical thickness (ROI analysis with data extracted from FreeSurfer), which includes a larger number of voxels in each region measured by the vertex-by-vertex analysis, we observed increased thickness in the right posterior cingulate cortex, including the isthmus cingulate (which is a narrow cortical area that connects the posterior end of the cingulate gyrus with the parahippocampal gyrus), and in the right and left lateral orbitofrontal cortex as well as decreased cortical thickness in the left paracentral and posterior cingulate and in the right temporal pole in the ASD group compared to controls (Supplementary Image 3; Table 5).

**Table 5:** Spatially distributed patterns of differences in cortical thickness in individuals with Autism spectrum disorder compared with controls—ROI analysis.

**Lobe**	**Region**	**Side**	**Centroid MNI coordinates**		
			**x**	**y**	**z**
		**ASD**>**Controls**			
Frontal	Lateral orbito-frontal	L	28.96	−12.24	53.54
	Lateral orbito-frontal	R	21	38	−19
Limbic	Posterior cingulate cortex/Isthmus cingulate	R	9	−39	14
		**ASD**<**Controls**			
Temporal	Temporal pole	R	42	21	−35
Limbic	Posterior cingulate	L	−7	−41	30
Other	Paracentral	L	−8	−32	69

*L, left; R, right. p < 0.05 (multivariate analysis with Bonferroni correction) for all the areas presented in the table*.

The ROI analysis showed significantly increased cortical surface area only in the right anterior cingulate (*p* = 0.019, multivariate analysis with Bonferroni correction).

We found also increased gyrification index in the postcentral, precentral, superior parietal, and supramarginal regions of both hemispheres, in the right frontopolar and middle frontal regions, and in the left paracentral region (Table 6).

**Table 6:** Spatially distributed patterns of differences in the gyrification index in individuals with Autism Spectrum Disorder compared with controls—ROI analysis.

**Lobe**	**Region**	**Side**	**Centroid MNI coordinates**		
			**x**	**y**	**z**
		**ASD**>**Controls**			
Frontal	Frontopolar	R	21	29	−23
	Middle frontal	R	63	8	37
Parietal	Superior parietal	L	−28.43	−65.42	20.57
	Superior parietal	R	28	−63	52
	Supramarginal	L	−36.5	−43.67	9.21
	Supramarginal	R	42	−38	32
	Paracentral	L	−8	−32	69
Central	Postcentral	L	−13.68	−18.60	52.44
	Postcentral	R	−30.83	17.08	42.62
	Precentral	L	8.59	11.93	64.19
	Precentral	R	−10.97	7.58	66.56

*L, left; R, right. p < 0.05 (multivariate analysis with Bonferroni correction) for all the areas presented in the table*.

#### Subcortical volumes

We found no differences between groups in the volumes of the amygdala, hippocampus, thalamus, or caudate.

### Functional connectivity

**Figure 4:**
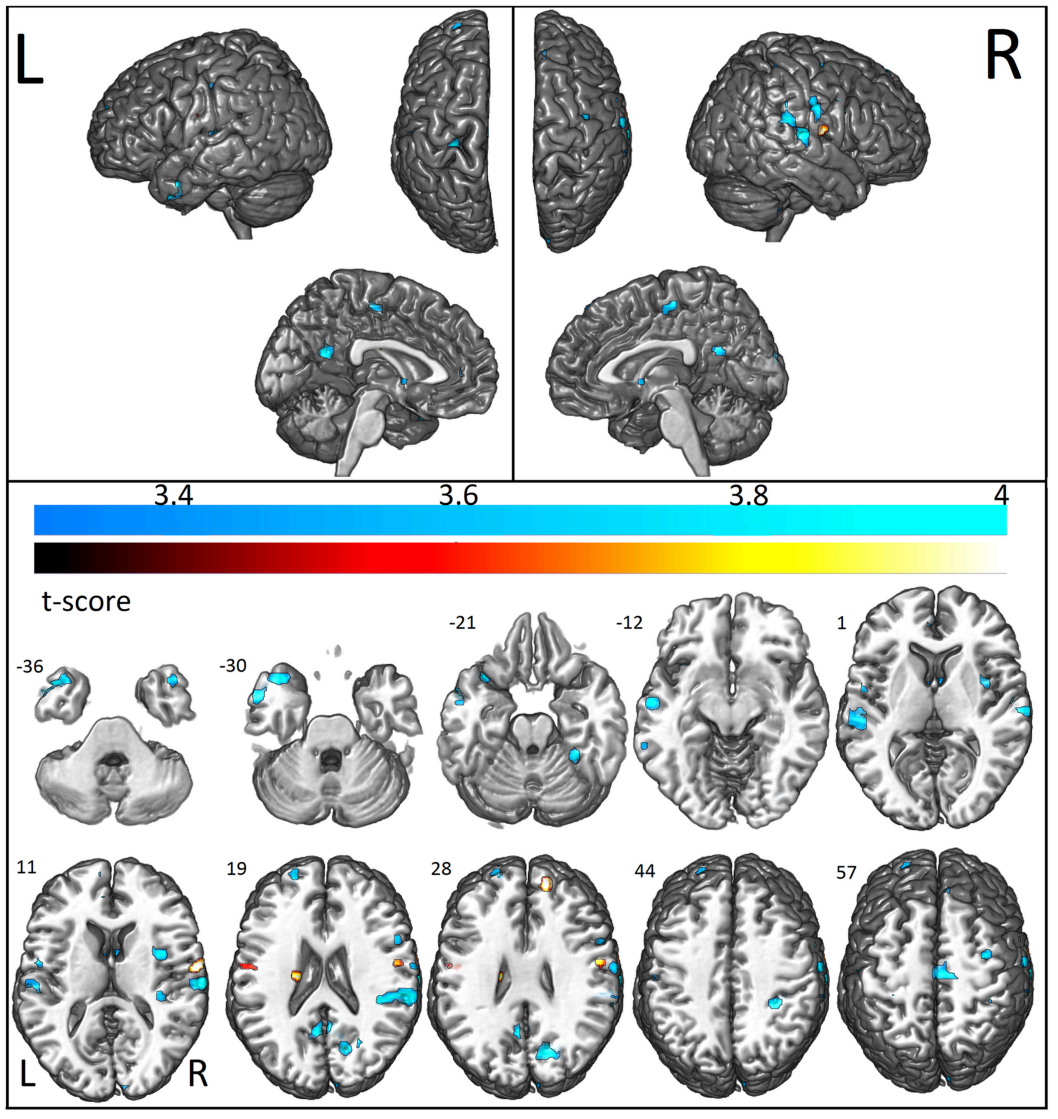
Areas with decreased (cool colormap) and increased (hot colormap) functional connectivity measurements in patients when compared to controls. In shades of blue (cool colormap), regions with maximum decreased functional connectivity (union of all seeds results, two sample *t*-test *p* < 0.001 clusters with at least 10 voxels) in patients compared to controls. In the hot colormap (black to yellow), regions of increased functional connectivity (two sample *t*-test, *p* < 0.001, cluster with at least 10 voxels).

We first examined the FC patterns of the PCC, which is part of the DMN. Relative to the control group, ASD patients showed reduced FC with the PCC (i.e., between the posterior part of the DMN and other areas of the brain) which was more pronounced in the left hemisphere, including the middle temporal gyrus, inferior, and superior frontal gyrus, and anterior and posterior cingulate. Decreased connectivity was also observed in other regions outside the DMN: the right cerebellum, cuneus, and caudate (Figure 4). Increased FC in areas of the DMN occurred only in the right middle frontal gyrus. Outside the DMN regions, increased connectivity was present in the left caudate (Figure 4).

The analysis of the additional seed positions as described in Methods, showed decreased FC in ASD patients between the left amygdala and right claustrum, inferior parietal lobule, postcentral gyrus, cingulate gyrus, precentral gyrus, inferior frontal gyrus, middle frontal gyrus, and left postcentral gyrus; between the left anterior frontal region and the right superior frontal gyrus; between the left anterior hippocampus and bilateral temporal, right insula, and left precentral regions; between the left temporal pole and the left temporal and parietal, right temporal, frontal, parietal, and occipital regions (Figure 4; Table 7). Increased FC was observed between left amygdala and right superior frontal gyrus, and between the middle frontal regions and bilateral pre- and postcentral gyrus (Figure 4; Table 7).

**Table 7:** Areas of significantly decreased and increased connectivity in patients with ASD in comparison with a group of healthy individuals.

**Seed region**	**Voxels**	**Area**	**Side**	**T score**	**MNI Coordinates**
**AREAS OF DECREASED FUNCTIONAL CONNECTIVITY IN PATIENTS WITH ASD**					
PCC^*^	47	Middle temporal gyrus	Left	4.66	−54 5 −26
PCC	56	Cuneus	Right	4.64	15 −70 25
PCC	83	Inferior frontal gyrus	Left	4.49	−39 17 −26
PCC	35	Posterior cingulate	Left	4.25	−6 −55 28
PCC	33	Superior frontal gyrus	Left	4.01	−21 59 25
PCC	20	Caudate	Right	3.82	3 2 −2
PCC	14	Cerebellum, Posterior lobe	Right	3.70	45 −37 −44
PCC	12	Anterior cingulate	Left	3.50	−6 44 13
Left amygdala	44	Insula	Right	4.47	36 2 13
Left amygdala		Claustrum	Right	3.57	27 8 19
Left amygdala	172	Inferior parietal lobule	Right	4.30	53 −31 22
Left amygdala		Postcentral gyrus	Right	3.83	53 −19 13
Left amygdala	44	Cingulate gyrus	Right	4.20	30 −34 40
Left amygdala	33	Precentral gyrus	Right	4.08	36 −4 55
Left amygdala	21	Inferior frontal gyrus	Right	3.89	54 11 25
Left Amygdala	44	Middle frontal gyrus	Right	3.88	3 −16 52
Left Amygdala	12	Postcentral gyrus	Left	3.44	−57 −15 43
Left ant. frontal	15	Superior frontal gyrus	Right	3.61	9 41 55
Left ant. hippocampus	84	Superior temporal gyrus	Left	4.20	−54 −28 4
Left ant. hippocampus		Transverse temporal gyrus	Left	4.03	−54 −19 10
Left ant. hippocampus	54	Superior temporal gyrus	Right	4.08	66 −19 10
Anterior hippocampus	33	Insula	Right	3.92	36 −28 13
Left ant. hippocampus	12	Precentral gyrus	Left	3.66	−54 −1 10
Left ant. hippocampus	10	Inferior frontal gyrus	Right	3.51	66 14 28
Left temporal pole	50	Postcentral gyrus	Left	4.31	−27 −28 67
Left temporal pole		Inferior parietal lobule	Left	3.45	−30 −34 58
Left temporal pole	34	Middle temporal gyrus	Left	4.25	−57 −10 −11
Left temporal pole	34	Cerebellum, Anterior lobe	Right	4.25	30 −40 −20
Left temporal pole		Parahippocampal gyrus	Right	3.49	27 −25 −20
Left temporal pole	28	Medial frontal gyrus	Right	4.25	12 −19 58
Left temporal pole	47	Superior temporal gyrus	Left	4.14	−48 8 −32
Left temporal pole	68	Postcentral gyrus	Right	4.00	63 −10 31
Left temporal pole	22	Superior temporal gyrus	Right	3.84	42 17 −38
Left temporal pole	72	Posterior cingulate	Right	3.83	3 −52 22
Left temporal pole	28	Cuneus	Right	3.83	21 −76 28
Left temporal pole		Precuneus	Right	3.57	24 −67 25
Left temporal pole	20	Middle frontal gyrus	Right	3.74	63 8 37
Left temporal pole	11	Postcentral gyrus	Left	3.69	−54 −4 13
**AREAS OF SIGNIFICANTLY INCREASED FUNCTIONAL CONNECTIVITY IN PATIENTS WITH ASD**					
PCC	22	Caudate	Left	4.13	−18 −16 25
PCC	10	Middle frontal gyrus	Right	3.60	45 8 61
Left amygdala	28	Superior frontal gyrus	Right	4.78	15 53 28
Bil. medial frontal region	74	Postcentral gyrus	Right	4.04	63 −7 13
Bil. medial frontal region		Precentral gyrus	Right	3.51	54 −4 31
Bil. medial frontal region	32	Precentral gyrus	Left	3.40	−51 −10 25
Bil. medial frontal region		Postcentral Gyrus	Left	3.29	−60 −7 22

* *PCC, Posterior Cingulate Cortex bilaterally (posterior aspect of the DMN). Ant, anterior; Bil, Bilateral; All regions in the table had p < 0.001 (two-sample t-test), cluster with at least 10 voxels*.

### Imaging and clinical scores

#### Cortical thickness and symptomatology

**Figure 5:**
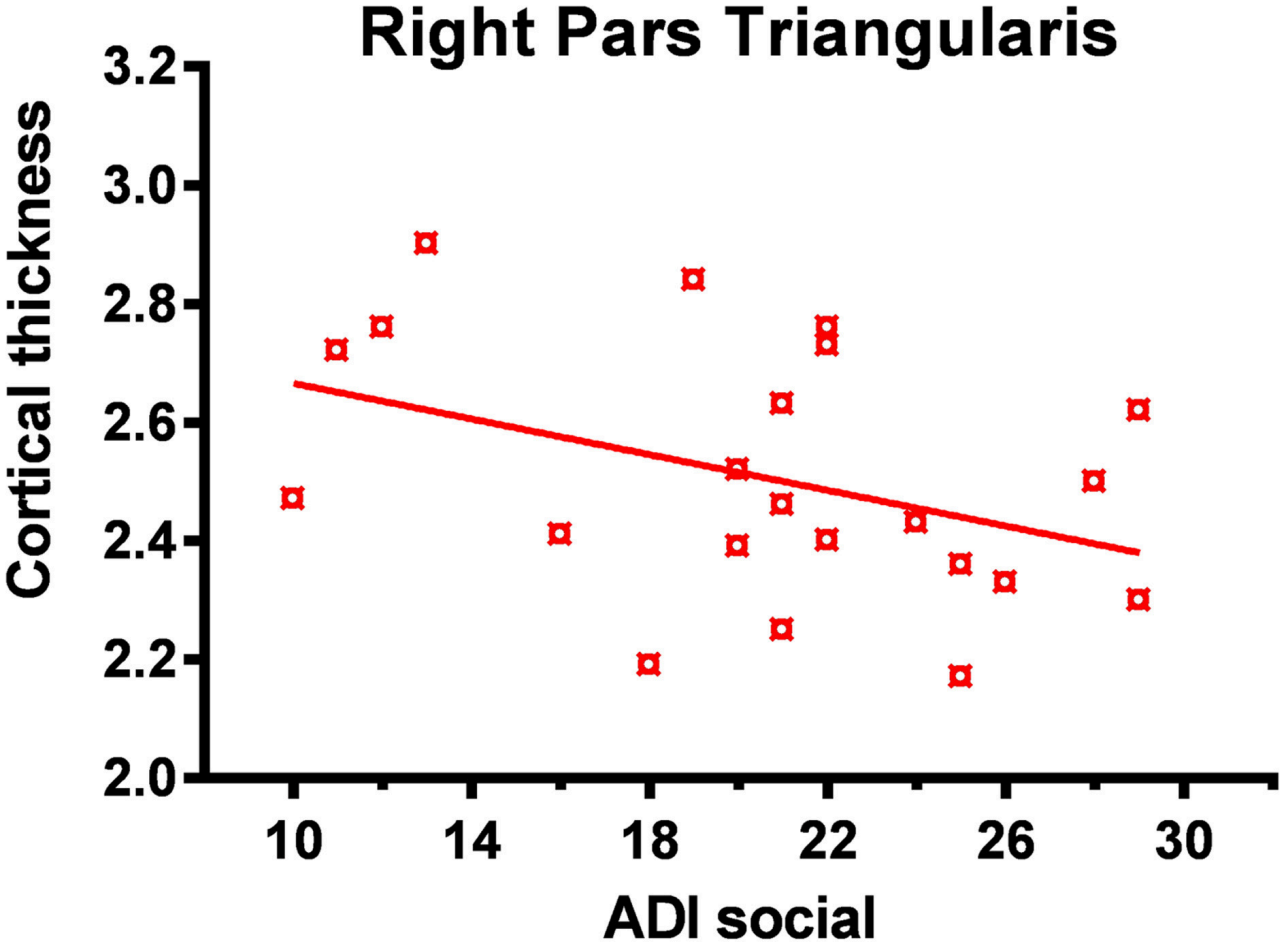
Reduced cortical thickness (from FreeSurfer ROI analysis) in the right inferior frontal lobe correlated with higher social impairment. In the ASD group reduced cortical thickness in the right pars triangularis was associated with greater social impairment as measured by the ADI-R (Autism Diagnostic Interview-Revised) social domain.

**Figure 6:**
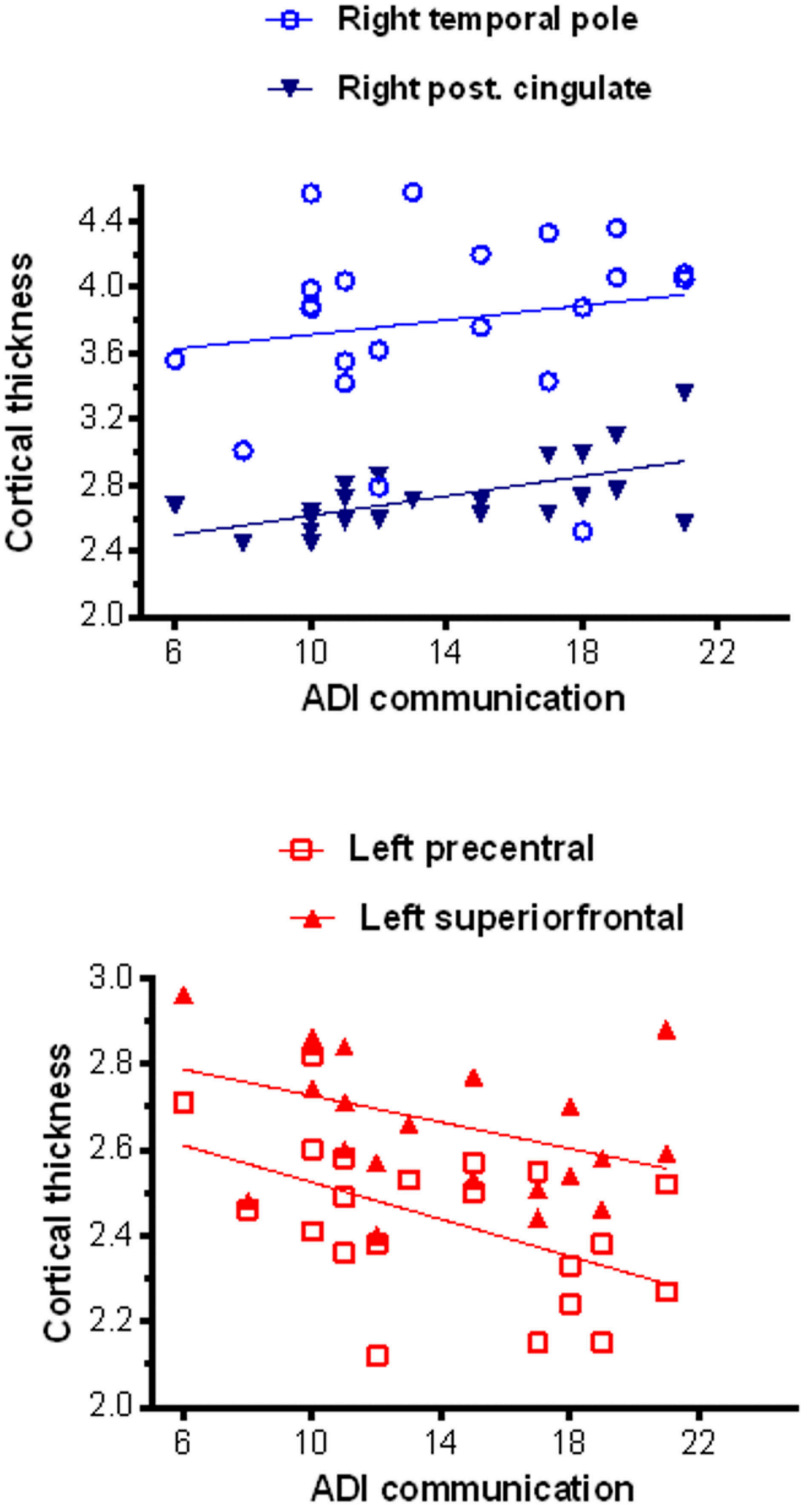
Correlations between cortical thickness (from FreeSurfer ROI analysis) and communication scores on the ADI-R. In the ASD group thicker cortices in the right temporal pole and right posterior cingulated were positively associated with greater communication impairment as measured by the ADI-R Autism Diagnostic Interview-Revised) social domain **(Top)**, while thinner cortices in the left precentral and superior frontal regions correlated with greater communication impairment as in the ADI-R social domain **(Bottom)**.

Significant correlation (corrected for age and total IQ) was found in the right pars triangularis (part of the lateralized fronto-parietal components of the DMN) (73), where reduced cortical thickness was associated with more impaired scores in the social domain of the Autism Diagnostic Interview-Revised (ADI-R) (*r* = −0.63; *p* < 0.001) (Figure 5). A significant negative correlation (*r* = −0.52; *p* = 0.02) was also found between cortical thickness in the left precentral and superior frontal regions (areas of the executive control and sensorimotor component of the DMN) (73) with communication scores on the ADI-R (Figure 6). Reduced cortical thickness in these areas was associated with more severe scores on the ADI-R communication domain. Thicker cortices in the right temporal pole (*r* = 0.56; *p* = 0.01) and posterior cingulate (*r* = 0.50; *p* = 0.03) were associated with greater communication impairment as measured by the ADI-R communication domain (Figure 6). We found no correlations between the scores on the restrictive and repetitive behaviors (RRIB) domain of ADI-R and structural images.

#### Functional connectivity and symptomatology

There was a trend for significant association (that did not survive Bonferroni correction) between stronger connectivity indexes from PCC to the right temporal pole (*p* = 0.09) and left anterior hippocampus (*p* = 0.10) with worse symptom severity in the social domain on the ADI-R, controlling for age and total IQ. We found no correlations between the scores on the RRIB domain of ADI-R and FC.

### Overlap of abnormalities across modalities

**Figure 7:**
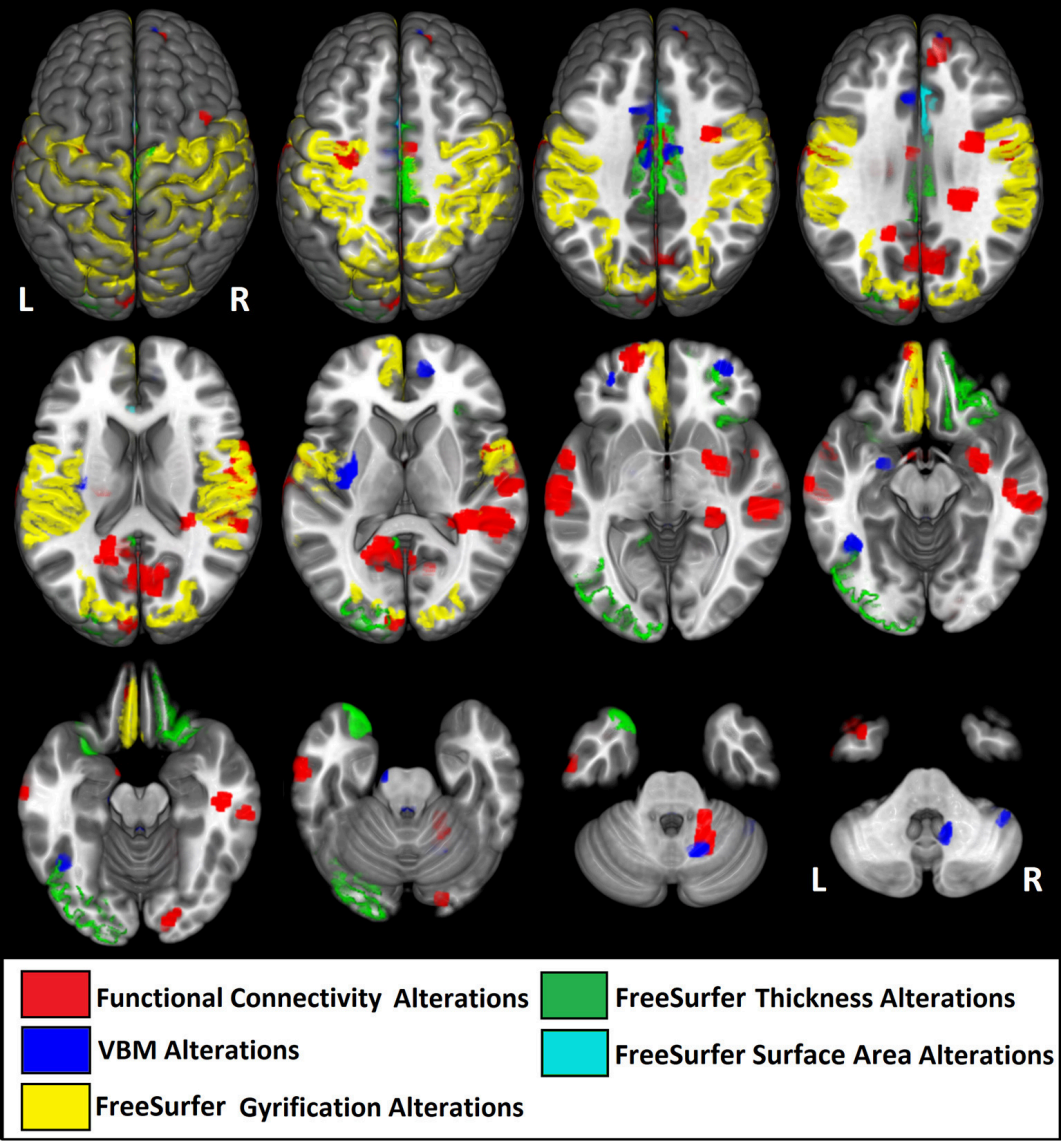
Illustrative figure showing anatomical localization of abnormalities in functional connectivity (in red), voxel-based morphometry (VBM, in blue), and FreeSurfer ROI analysis of gyrification index (in yellow), cortical thickness (in green), and surface area (in light blue). The areas indicated in this figure do not correspond to the maximum voxel statistical location, but rather the sub anatomical regions with significant differences in patients with high functioning autism compared to controls. See Tables 2–7 for the centroid MNI coordinates of maximal abnormalities and Table 8 for a summary of the location of increased and decreased changes as compared to controls.

**Figure 8:**
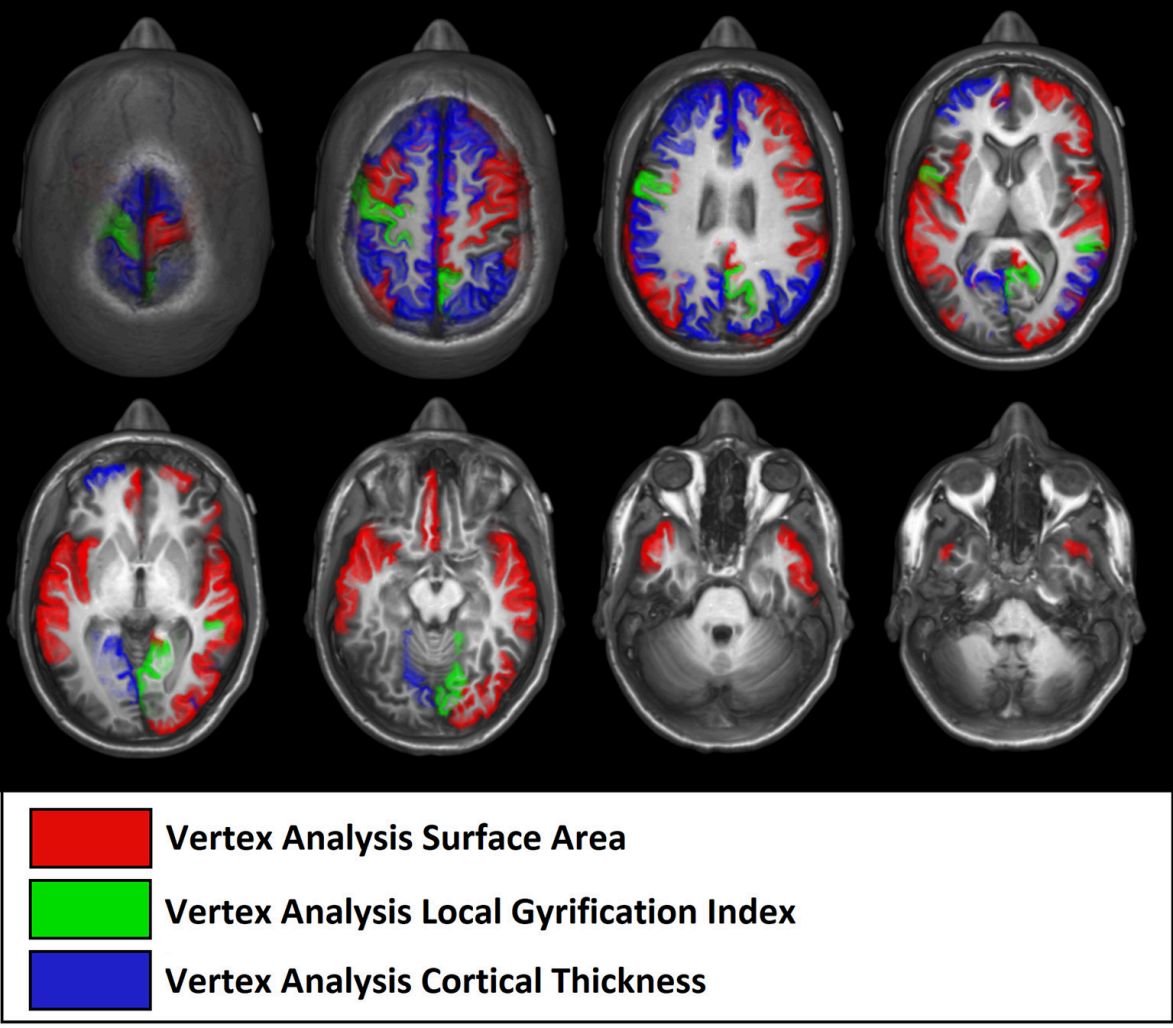
Illustrative figure showing anatomical localization of abnormalities of FreeSurfer vertex-wise analyses of surface area (in red), gyrification index (in green), and cortical thickness (in blue). The areas indicated in this figure do not correspond to the maximum voxel statistical location, but rather the sub anatomical regions with significant differences in patients with high functioning autism compared to controls. See Figure 3 and Table 4 for the location of maximal abnormalities and Table 8 for a summary of the location of increased and decreased changes as compared to controls.

The percentage of coincident maximum voxels abnormalities between resting-state FC and abnormal gray matter on VBM was <3%. However, we found a close localization of the FC abnormalities and GM reduction on VBM and changes in cortical thickness in FreeSurfer ROI analysis (Figure 7) and vertex-wise analysis (Figure 8) in cingulate gyri of both hemispheres, left parahippocampal gyrus, postcentral gyrus, amygdala, and claustrum; right middle and superior frontal gyri, temporal pole, and cerebellum (Table 8).

**Table 8:** Sub-regional overlap of abnormalities across structural and functional MRI modalities.

**Lobe/Region**	**Area**	**GM/VBM**		**Cortical Thickness**				**Cortical Surf. area**				**Gyrification Index**				**FC of DMN**	
				**FS-VxV**		**FS-ROI**		**FS-VxV**		**FS-ROI**		**FS-VxV**		**FS- ROI**			
		**Left**	**R**	**Left**	**R**	**Left**	**R**	**Left**	**R**	**Left**	**R**	**Left**	**R**	**Left**	**R**	**Left**	**R**
Frontal	Orbito-Frontal					↑	↑	↑									
	Cingulate	↓	↓		↓	↓	↑	↑	↑		↑					↓ ↑	↓
	Sup. Frontal		↓	↓	↓			↑	↑							↓	↓ ↑
	Middle Frontal		↓	↓				↑	↑						↑		↓ ↑
	Inf. Frontal/Pars triang.								↑							↓	↓
	Frontopolar														↑		
Temporal	Pole	↓				↓										↓	
	Sup. Temp. sulcus/G.	↓		↓			↓	↑								↓	↓
	Middle Temp. G.	↓			↓			↑	↑				↑			↓	↓
	Inf. Temp. G							↑	↑								
	Fusiform G	↓															
	Parahippocampus	↓														↓	
	Amygdala	↓														↓	↓
	Hippocampus															↓	
	Insula							↑									↓
	Claustrum	↓															
	Caudate															↑	↓
Central	Paracentral	↓		↓		↓	↓		↑			↑		↑			
	Precentral			↓	↓			↑	↑			↑		↑	↑	↓ ↑	↓ ↑
	Postcentral	↓		↓ ↑	↓			↑						↑	↑	↓ ↑	↓ ↑
Parietal	Supramarginal			↓					↑					↑	↑		
	Sup. parietal			↓	↓								↑	↑	↑		
	Inf. Parietal			↓	↓			↑								↓	↓
	Precuneus			↓				↑	↑				↑				
Occipital	Lingual G.			↓									↑				
	Lateral Occip.								↑								
	Cuneus																↓
Cerebellum	↓	↓ ↑														↓	↓

*↓, Decreased ↑, increased. GM, gray matter; VBM, voxel-based morphometry; FS-VxV, FreeSurfer voxel by voxel analyses; FS-ROI, FreeSurfer voxel region of interest (ROI) analyses; FC, functional connectivity; DMN, default mode network, G, gyrus*.

Note that the lack of correspondence between the maps presented in Figures 2, 3 and the results in Figures 7, 8, is because in Figures 2, 3 the maps show the most statistically significant clusters of abnormalities while in Figures 7, 8 the areas indicated do not correspond to the maximum voxel statistical location, but rather the sub-anatomical regions with significant differences (therefore, much larger than in Figures 2, 3).

GM atrophy determined by VBM showed a closer anatomical relationship with reduced FC than surface measures by FreeSurfer. Interestingly, areas with decreased GM volume (middle and superior temporal gyri, parahippocampus, and amygdala/uncus, all in the left hemisphere) that correlated with increasing age in patients had reduced FC (see Table 3).

## Discussion

The diversity of neuroimaging results are likely explained by the heterogeneous nature of ASD, both among the subgroups within the spectrum, the variable comorbidities and on an individual level across the lifespan (15, 29, 35, 43, 44, 50, 57–60, 63, 67, 84–86, 107, 135). The individual differences in functional and structural organization, the idiosyncratic ASD connectivity and cortical atrophy maps, which change over the maturation of central nervous system, are themselves the core features of ASD, although its pathophysiological basis remains undetermined (13, 15). These findings underscore the need to address both age and severity when investigating functional and structural neuroimaging in ASD (15). Every imaging technique, both regarding acquisition and post-processing have their limitations and advantages and are in constant improvement of the quality of acquisition (better hardware) and algorithms of post-processing. These facts make it difficult to compare studies over the years. The use of multimodal imaging in a single study, in a similar age range and severity of symptoms, may provide a better description of the altered brain connectivity and structural changes, and its relationship with behavioral changes, than one imaging method alone. However, several multimodal studies have been performed with some contradictory findings, which by itself justify further studies (6, 15, 29, 35, 37, 43, 44, 50, 57–60, 63, 67, 84–86, 107, 135).

Different from most studies that focused on a single technique (27, 28, 108–112) or low functioning autism (113), or using a heterogeneous group of patients (114), our multimodal imaging investigation showed abnormalities across brain measures in young adults and adolescents with high-functioning autism. We showed reduced cortical thickness, increased cortical surface and increased gyrification, as well as abnormal functional connectivity, mostly co-localized in areas that are important hubs of the default mode network and other regions frequently linked to socio-emotional processing, such as cingulum, amygdala, insula, and temporal pole. Overall, our findings suggest aberrant functional connectivity involving a network of altered cortical structure.

We combined structural and functional connectivity analyses to detect complex brain abnormalities and to investigate how these alterations are related to each other and symptom severity in a group of individuals with high functioning autism. We observed that patients with ASD had decreased FC compared to controls between the PCC and anterior medial prefrontal cortex and left superior temporal cortex (temporal pole), both regions part of the DMN. Patients also exhibited greater diffuse subtle GM atrophy related to increasing age (in the VBM analysis), more pronounced in left temporal regions (temporal pole, middle temporal gyrus, parahippocampal gyrus, and uncus). In addition, we showed areas of abnormal cortical structure, combining thinning, and thickening, increased surface area and gyrification index in different areas of the brain, involving frontal, parietal, and temporal areas that had abnormal FC. Overall these structural and functional abnormalities involved areas linked to: (a) visual processing and analysis of logical order of events (lingual gyrus), (b) encoding visual memories (temporal and posterior cingulate areas), (c) areas related to language, memory and emotion processing (temporal pole, middle temporal, parahippocampus, and uncus), (d) areas of the executive control component of the DMN, which has been associated with performance of executive functional tasks (anterior and posterior cingulate cortex, left middle temporal, inferior, and superior frontal gyrus), (e) areas of the sensorimotor component of the DMN (anteromedial prefrontal cortex and bilateral pre- and postcentral gyrus), (f) areas of the lateralized fronto-parietal components of the DMN related to executive and language functions (reduced cortical thickness in left frontal regions), and (g) areas of the auditory component of the DMN (temporal and parietal areas) (73, 115). In addition, more severe scores on the communication domain of the ADI-R were associated with increased cortical thickness in the right temporal and posterior cingulate gyrus, and there was a trend for worse symptoms in the social domain of the ADI-R to be associated with stronger connectivity between posterior cingulate cortices (DMN) and temporal regions (areas of the Auditory component of the DMN) (71–73).

Our findings taken together indicate that young adults and adolescents with high functioning autism present complex, subtle morphological cortical changes that may reflect different stages of neurogenesis, combined with aberrant connectivity within and outside the DMN.

### Structural abnormalities

To date, neuroimaging studies in ASD have mainly investigated either cortical volume or cortical thickness in isolation, and combined measures of surface area and gyrification with functional data remain scarce (4). Studies in adults with ASD typically show cortical thickening of the frontal cortex (6, 116, 117), whereas the cortical thickness of the temporal lobe has been reported as increased or decreased in patients with ASD (118).

Abnormal brain structure has been reported with great variability in individuals with ASD, both enlargement, and reduction of the GM (40, 46, 119). However, this variability is probably due to the highly heterogeneous age of the patients (from children to adults) and various phenotypes (5, 120). It is believed that in ASD there is a disruption of the time course of brain development and this could be the explanation for the detection of specific increased areas in children during an early phase of development and reduced areas (atrophy) in adults (40). Our findings, which included only ASD individuals with total IQ > 85, confirm this theory and add further evidence about specific types of abnormal cortical shape and volume in association to functional abnormalities. Another key aspect of our results is that we used multimodal imaging measures in the same patients to certify that the abnormalities are present across brain measures, different from most studies so far that focused on a single technique.

Volumetric studies of ASD in earlier MRI studies showed increased volumes in left frontal and temporal lobes across the 2- to the 11-year-age range (121) and in the dorsolateral prefrontal and medial frontal cortex in patients aged 2–5 years (122). A meta-analysis showed that brain size in autism was slightly reduced at birth, increased within the first year of life, and within normal range by adulthood (123). However, it is difficult to compare these studies since the methodologies for cortical volume measurements varied significantly (manual volumetry, VBM with different versions of SPM software, cortical thickness). Also, earlier studies used images with lower MRI field strength (1.5 T) as compared to the higher fields (3T MRI) and higher resolution images used in more recent studies. More recent versions of SPM software (http://www.fil.ion.ucl.ac.uk/spm/software/) have substantial algorithmic enhancements with more sophisticated registration models compared to previous versions and thus, making it difficult to compare earlier studies with more recent ones (45, 124). These aspects and the fact that our patient's ages ranged from 14 to 25 years (mean: 17.4 years) may explain why our VBM analyses (excluding the cerebellum and brainstem) did not show areas of increase GM and showed GM atrophy mainly in temporal and frontal areas.

VBM and FreeSurfer cortical measures use quite different methods and are expected to yield different results as we showed here. Our intention was not to compare these two methods, but rather to expand the search for structural changes in these patients in a multimodal way. We believe that these two techniques added information and were not redundant. VBM performs voxel-wise statistical analysis on smoothed (modulated) normalized segments (90, 124). VBM is a statistical parametric mapping of segmented tissue density and compares the local concentration of gray matter between two groups of subjects (90, 124). The interpretation of gray matter concentration or density depends on the preprocessing steps used (90, 124). However, VBM is a whole-brain unbiased, objective technique, with very reproducible results in similar circumstances (of image quality and software version), providing great sensitivity for localizing small-scale, regional differences in gray matter concentration (90, 124, 125). In addition, more rigorous methods for correction for multiple comparisons will reduce the false positives but also reduce the pickup rate of true positives.

FreeSurfer uses the cortical geometry to do inter-subject registration, which appears to have a much better matching of homologous cortical regions than other volumetric techniques. FreeSurfer allows measuring the two components of volume separately (thickness and surface area). These two measures are not similar and do not necessarily change in parallel as will be discussed below (37). FreeSurfer uses the white matter surface geometry for registration, which is completely independent to GM atrophy; therefore, GM alterations will not result in different registrations (92–94, 99). Therefore, one should not expect a total overlap between findings with VBM and FreeSurfer in the same group of subjects, as it was in this study.

Using FreeSurfer, we found significant differences in cortical thickness of ASD patients over frontal regions (superior, middle frontal regions, pars orbitalis) and temporal lobes (right temporal pole). This finding is consistent with previous reports suggesting that people with ASD have differences in frontal lobe neuronal integrity, function, anatomy, and connectivity. Furthermore, it has been suggested that individuals with ASD have a delay in frontal lobe maturation and that abnormalities in frontal lobe development may underlie some of the social impairments reported in people with ASD (39, 122, 126), which was corroborated by our results.

Cortical surface areas are usually, but not necessarily, increased (as illustrated in Table 7) in regions with reduced cortical thickness, which is biologically explained by the consequent increase in sulcation of the cortical mantle (i.e., with atrophy the sulci became deeper, thus increasing the area) (37). Therefore, explaining our finding of increased cortical surface areas coinciding with the regions with reduced cortical thickness described above, as well as in the pre- and post-central, orbitofrontal, posterior cingulate, inferior parietal, temporal lobes, and insular regions. However, cortical thickness and surface area measurements represent distinct aspects of the cortical architecture and may represent different early neurodevelopmental pathologies (37, 127, 128). Cortical thickness measurements appear to reflect the number of neurons within cortical minicolumns (mainly related to intermediate progenitor cells), while cortical surface area measurements may be related to the number of cortical minicolumns (mainly related to radial unit progenitor cells), according to the radial unit hypothesis (5, 37, 117, 127–129). Our findings suggest that, in addition to the well-documented early brain overgrowth in ASD, there is probably an arrested growth during late childhood, followed by accelerated regionally specific thinning during adolescence and young adulthood. More specifically, the present results complement earlier findings of thinner cortices in adults with ASD (5, 130–132).

We found increased gyrification in temporal, parietal, and frontal areas in ASD, supporting previous studies that indicate that these are the core areas in ASD and are probably related to abnormalities in visual-spatial attention, selective attention, and visual-motor learning as well as in the mirror neuron system (133, 134). Gyrification represents the amount of cortex within sulcal folds in the surrounding area of measurement and is computed as the ratio between the surface of the outer surface of the brain and the surface of the corresponding area on the GM (pial) surface (37, 95, 129), which reflects an early developing process. It is believed that the brain in ASD goes through a stage of accelerated expansion during early childhood, and consequently, ASD patients are expected to have an increase in cortical folding to accommodate an increasing brain surface into the skull (37, 127). A closer inspection of Figure 3, reveals that the areas (representing the points of maximal statistical scores) of reduced cortical thickness, increased cortical surface areas and increased gyrification areas have a similar distribution in our group of young adults and adolescents with ASD.

Abnormalities found in our analysis could be implicated in the core behaviors often impaired in ASD: social and communication (medial frontal region, anterior cingulate) and repetitive and stereotyped behavior (medial and lateral orbitofrontal region).

### Resting-state functional connectivity

Findings from most studies have continued to support the broad notion that, overall, individuals with ASD have poorer connectivity in regions spanning long distances in the brain compared to controls, whereas connectivity seems to be increased in local circuits (6, 47). However, findings amongst studies on FC in ASD do not overlap [some with increased (106) and others with decreased (51, 86, 135) connectivity in similar areas], in part due to different techniques used (i.e., seed analyses of predetermined areas, region of interest analyses, etc.) and heterogeneity of patient groups and age range, as occurs with the structural data discussed above (53, 107, 68). Others have reported decreased connectivity of the DMN in adolescents and adult patients with ASD (14, 51, 52, 54, 87), associated with more severe symptoms (135, 136). We found increased connectivity in the ASD group in the right middle frontal gyrus, and a trend for an association between the right temporal pole and left anterior hippocampus FC strength and ADI-R social score, indicating that worse symptom severity was associated with more connectivity in this region. Overall, our results are similar to those observed by Supekar et al. (106) about brain hyperconnectivity predicting symptom severity in ASD. Individuals with greater FC showed more severe social deficits, and they argue that this brain-behavior relationship suggests that aberrant FC may underlie social deficits, which are some of the hallmarks of ASD (28, 106). Our results add to the growing evidence that regional DMN under-connectivity may underlie the pathogenesis of patients' clinical deficits and go further by showing that seed-based analysis reveals the reduction in connectivity also in areas outside the DMN (amygdala, insula, and temporal pole), supporting that ASD is not only a condition of under- or hyper-connectivity but also of aberrant FC (13, 14, 27, 29, 35, 37, 54–56, 69, 137–140).

#### The role of the temporal pole

We found significant VBM cortical atrophy in ASD individuals when considering age, only in the left temporal lobe, including the left temporal pole. We also observed decreased FC in individuals with ASD between the left temporal pole and the remainder of left temporal and parietal regions. This region lies between the orbital frontal cortex and the amygdala, two of the region's most frequently linked to socio-emotional processing. The temporal pole is highly connected with the amygdala, hippocampus, parahippocampal gyrus, cingulate gyrus, orbitofrontal cortex, and the insula (141, 142). In addition, the temporal pole cortex extends topographically to the insula (ventrally) and the entorhinal cortex (medial-inferiorly) (142). The role of the temporal pole is key for various social and emotional functions, including mentalizing (theory of mind) (56, 66, 141, 143, 144). The impairment of theory of mind abilities is one of the most popular hypotheses about ASD (56, 66, 86, 144–146). Some studies using theory of mind tasks showed temporal pole activation (147–150), which give support to our interpretation of the temporal pole as a key node in ASD and social dysfunctions.

### Overlap between functional and structural findings

Our findings give further evidence that ASD is a network disorder, as revealed by the structural and functional abnormalities (112, 151). In a similar vein, Honey et al. observed that, although resting-state FC is variable and is often present between regions without direct structural linkage, its strength, persistence, and spatial statistics are nevertheless constrained by the large-scale anatomical structure of the human cerebral cortex (152).

We found no complete voxel overlap of areas of maximal GM reduction and areas of decreased connectivity in our patients, which is expected due to the different anatomical resolution between structural and functional images (original voxel sizes of 1 vs. 3 mm^3^, which became even more discrepant after spatial smoothing) and differences in post processing and analyses. However, close localization of the abnormalities was observed in cingulate gyri of both hemispheres, left parahippocampal gyrus, postcentral gyrus, amygdala, and claustrum, right middle, and superior frontal gyri, temporal pole, and cerebellum. Interestingly, GM atrophy determined by VBM showed a closer anatomical relationship with reduced FC than surface measures by FreeSurfer; particularly in areas with GM reduction in the left hemisphere that correlated with increasing age in ASD patients (middle and superior temporal gyri, parahippocampus, and amygdala/uncus). These differences may be explained by the distinct methods for quantification used by VBM and FreeSurfer, which may also reflect different biological substrates between GM volume vs. cortical thinning and cortical areas as discussed above. Nevertheless, our results support the notion that brain alterations in high functioning autism, although subtle and diffuse, converge into areas of structural and functional changes of higher order multisensory association cortex (58). Also, the lack of close correlation between cortical thickness and FC patterns [as also found in other diseases (100)] indicate that changes in cortical thickness or GM atrophy that are not severe enough to be seen on routine MRIs, do not impact directly on FC patterns. This observation is in line with studies of brain networks showing that structural and functional network communities rarely overlap; i.e., functional modules are not always directly connected anatomically [for review see (153)].

## Limitations

Limitations of our study include the potential effects of medication, a relatively small sample size that may have reduced statistical power and lack of information about puberty stages. However, the statistical significance of the results after correcting for multiple comparisons was remarkable. Our results cannot be generalized to younger and lower-functioning individuals with ASD since we studied a group that included only high functioning autism.

## Conclusion

We found cortical thinning and diffuse GM reduction, more pronounced in the left hemisphere, as well as decreased FC between the left hemisphere and PCC (posterior aspect of the DMN) in patients with high-functioning autism. Reduced cortical thickness in the right inferior frontal lobe correlated with higher social impairment, while thinner cortices in the left precentral and superior frontal regions and thicker cortices in the right temporal pole and posterior cingulated correlated with greater communication impairment.

The combination of these abnormalities might represent a neurobiological pattern of this end of the spectrum of autism disorders, indicating a network disorder and could help explain some of the core behaviors in ASD. We also believe that new techniques, such as cortical thickness measurements and surface morphometry could help to elucidate in more detail the patterns of abnormalities related to age and the neurodevelopmental process.

## Author contributions

AP and BC conducted data collection, data analyses and wrote the manuscript. AC, LP, TdR, IO, PD, and JdC contributed with study design and manuscript preparation and revision. J-CD contributed with study design, supervision, analyses of data, manuscript preparation and revision. FC contributed to study design and organization, supervision, funding, data analyses, manuscript preparation, and revision.

### Conflict of interest statement

The authors declare that the research was conducted in the absence of any commercial or financial relationships that could be construed as a potential conflict of interest.

## Supplementary material

Supplementary Image 1 Z-scored average connectivity maps of all seeds from both groups. With **(a)** we indicate controls' average maps, with **(b)**, patients' average maps. In (1), DMN maps, with seed on the posterior cingulated cortex; in (2) the seeds in the left temporal pole; in (3) with the seed on the left anterior hippocampus; in (4), with the seed on the left amygdala; in (5), the seed on the interhemispheric medial frontal gyrus. The slices in (1), (2), and (5) were MNI axial: −32, −12, 18, 48, 78, and in (3) and (4) were MNI axial: −26, −12, 18, 48, 78.

Supplementary Image 2 Areas of gray matter atrophy in voxel-based morphometry influenced by the age in patients with ASD. Gray matter atrophy determined by voxel-based morphometry, *p* < 0.001 clusters with at least 30 voxels.

Supplementary Image 3 Inflated surface maps showing areas with increased and decrease cortical thickness in ASD compared to controls using ROI analysis. There was an increased thickness in right posterior cingulate (red), and in the right (green) and left lateral orbitofrontal cortex (blue) as well as decreased cortical thickness in the left paracentral (pink), posterior cingulate (yellow), and in the right temporal pole (orange) in the ASD group compared to controls.
