# Supplementary figures and images for: Differences in Cortical Structure and Functional MRI Connectivity in High Functioning Autism
Source: Front Neurol. 2018 Jul 10;9:539. doi: 10.3389/fneur.2018.00539 (PMC6048242; doi:10.3389/fneur.2018.00539)

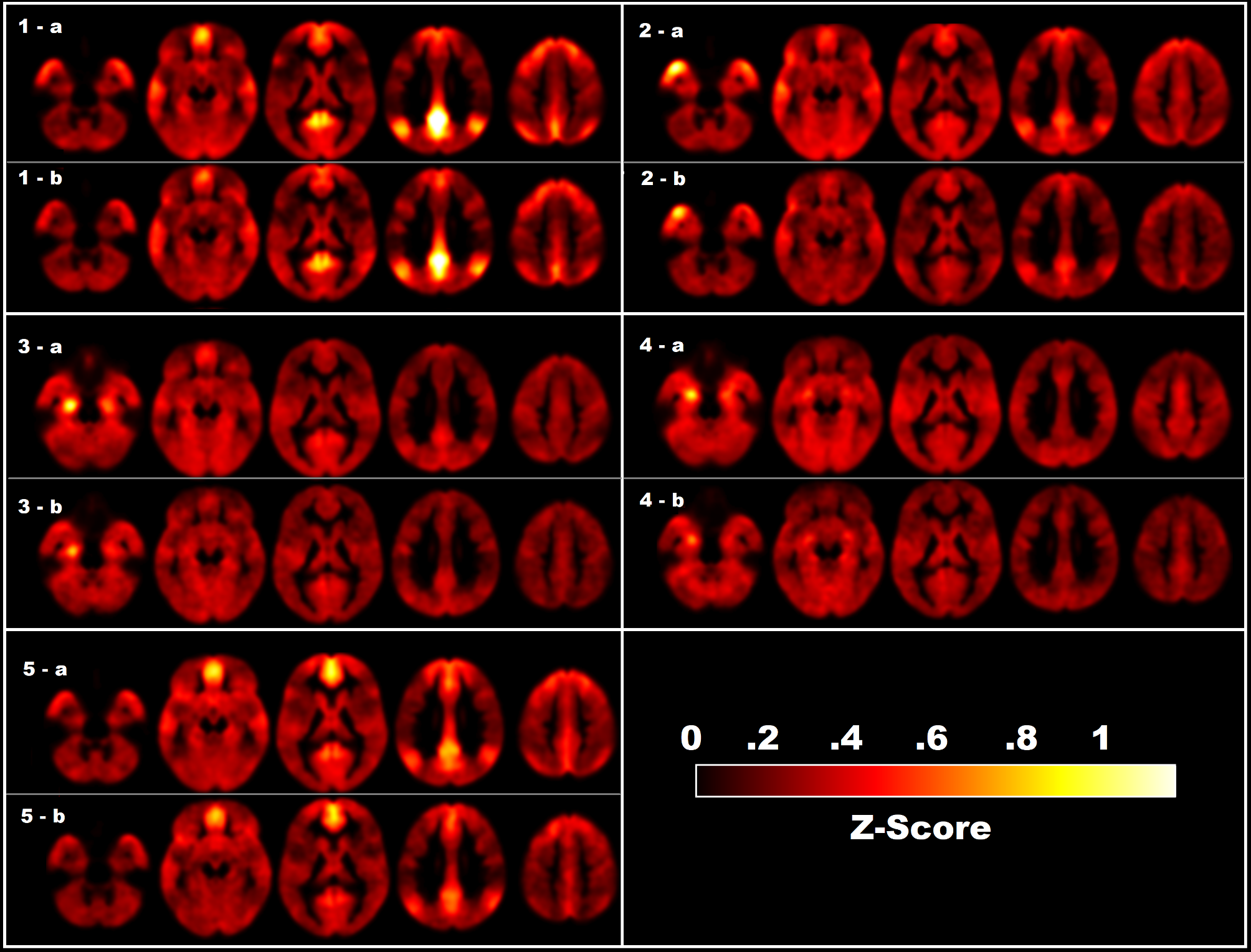

Supplement: Supplementary Image 1 — Z-scored average connectivity maps of all seeds from both groups. With (a) we indicate controls' average maps, with (b), patients' average maps. In (1), DMN maps, with seed on the posterior cingulated cortex; in (2) the seeds in the left temporal pole; in (3) with the seed on the left anterior hippocampus; in (4), with the seed on the left amygdala; in (5), the seed on the interhemispheric medial frontal gyrus. The slices in (1), (2), and (5) were MNI axial: −32, −12, 18, 48, 78, and in (3) and (4) were MNI axial: −26, −12, 18, 48, 78. [file Image_1.TIF]

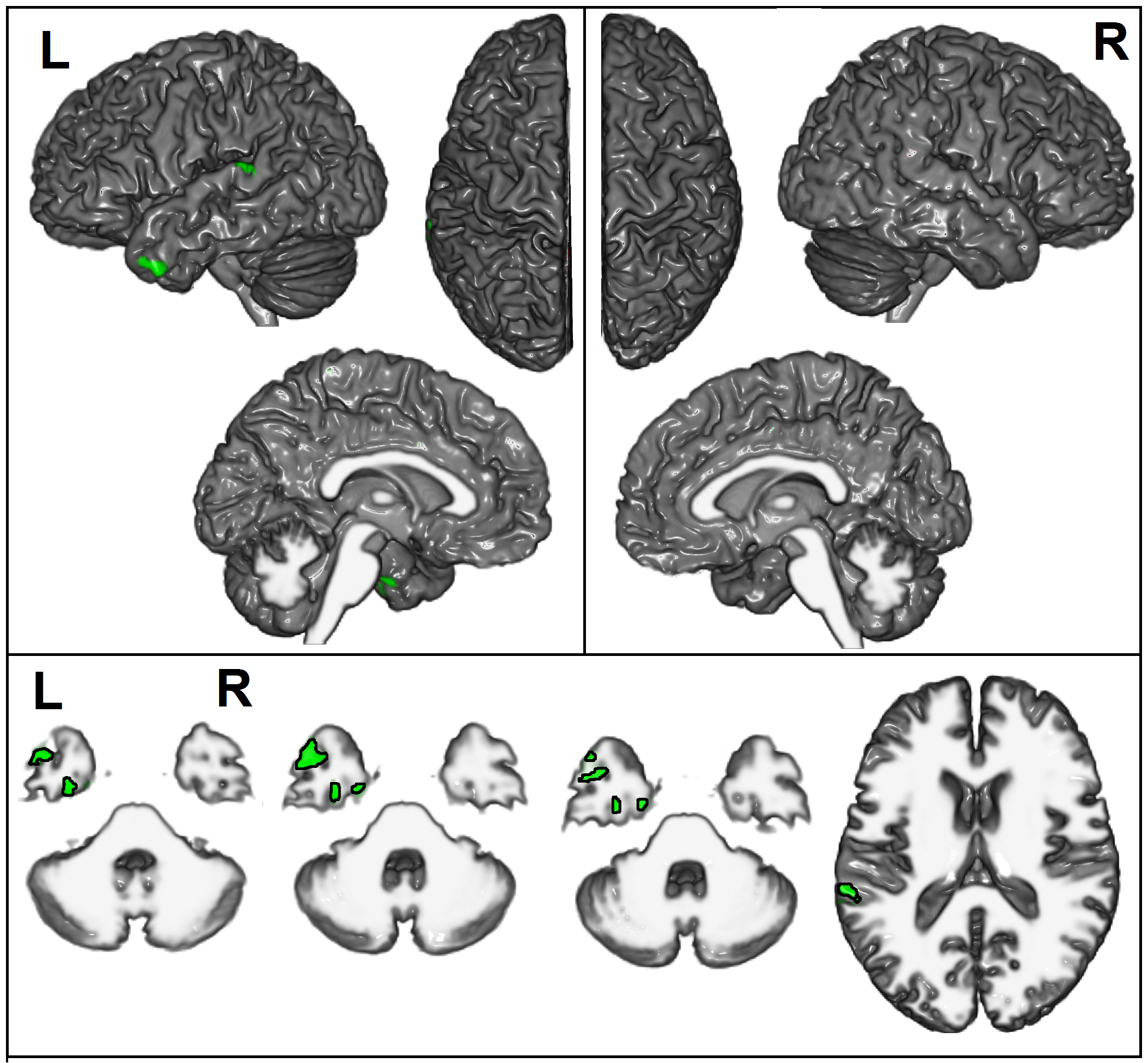

Supplement: Supplementary Image 2 — Areas of gray matter atrophy in voxel-based morphometry influenced by the age in patients with ASD. Gray matter atrophy determined by voxel-based morphometry, p < 0.001 clusters with at least 30 voxels. [file Image_2.TIF]

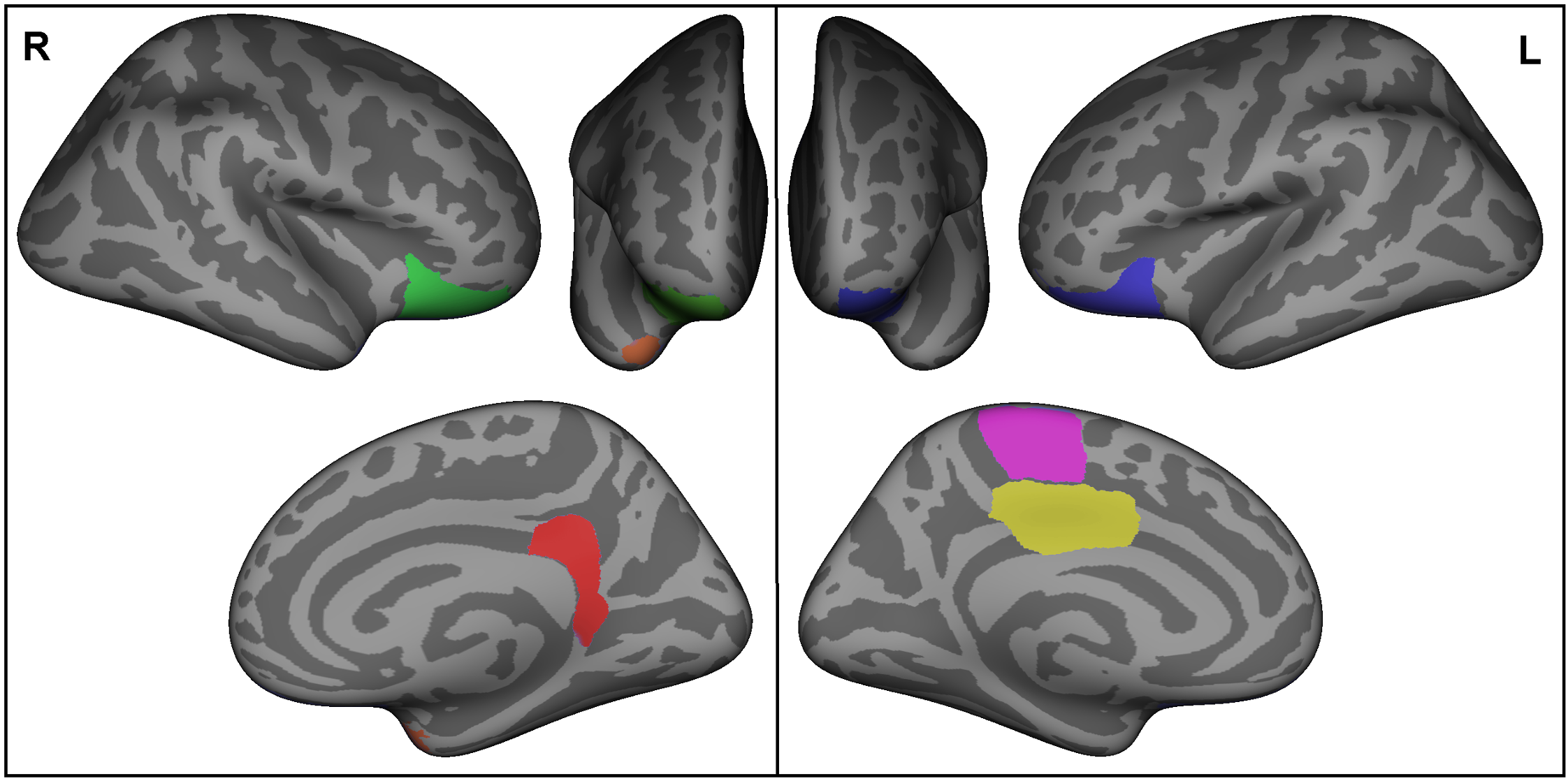

Supplement: Supplementary Image 3 — Inflated surface maps showing areas with increased and decrease cortical thickness in ASD compared to controls using ROI analysis. There was an increased thickness in right posterior cingulate (red), and in the right (green) and left lateral orbitofrontal cortex (blue) as well as decreased cortical thickness in the left paracentral (pink), posterior cingulate (yellow), and in the right temporal pole (orange) in the ASD group compared to controls. [file Image_3.TIF]
